# Development and validation in 500 female samples of a TP-PCR assay to identify *AFF2* GCC expansions

**DOI:** 10.1038/s41598-021-93473-5

**Published:** 2021-07-19

**Authors:** Cecília Silva, Nuno Maia, Flávia Santos, Bárbara Rodrigues, Isabel Marques, Rosário Santos, Paula Jorge

**Affiliations:** 1 Unidade de Genética Molecular, Centro de Genética Médica Jacinto de Magalhães (CGM), Centro Hospitalar Universitário do Porto (CHUPorto), Porto, Portugal; 2 Unidade Multidisciplinar de Investigação Biomédica (UMIB), Instituto de Ciências Biomédicas Abel Salazar (ICBAS), Laboratory for Integrative and Translational Research in Population Health (ITR) Universidade do Porto (UP), Porto, Portugal

**Keywords:** Clinical genetics, Genotype, Medical genetics, Genetics research

## Abstract

Over 100 X-linked intellectual disability genes have been identified, with triplet repeat expansions at the *FMR1* (FRAXA) and *AFF2* (FRAXE) genes being the causative agent in two of them. The absence of FRAXE pathognomonic features hampers early recognition, delaying testing and molecular confirmation. Hence, our laboratory uses a multiplex PCR-based strategy to genotype both FRAXA and FRAXE. However, *AFF2* expansions are missed giving rise to an uninformative result in around 20% of female samples. To rule out undetected expansions and confirm homozygosity Southern blot analysis is performed being labour- and resource-intensive. The aim of this study is to develop a timely and economic triplet-primed amplification (TP-PCR) screening strategy to size the *AFF2* GCC repeat and accurately assess homozygosity as well as pinpoint multiplex-PCR false negatives in female samples. In order to achieve this, validation was performed in a cohort of 500 females with a previous uninformative FRAXE PCR result. Interestingly, the presence of a T > C SNP (rs868949662), contiguous to the GCC repetitive tract, allows triplet primer binding in two additional repeats, increasing the discrimination power of the TP-PCR assay in heterozygous and homozygous samples. Twelve alleles outside the normal range were recognized: eight intermediate and four premutated, which seems relevant considering the rarity of the *AFF2* expansions. All genotypes are concordant with that obtained by Southern blotting, confirming this as a strict, reproducible and low-cost homozygosity screening strategy that enables the identification of small expanded alleles missed by the routine multiplex-PCR due to allele dropout. Overall, this assay is capable of spotting multiplex-PCR false negatives besides identifying alleles up to > 80 GCC repeats. Furthermore, the occurrence of intermediate repeat sizes with unexpected frequency, introduces new areas of clinical research in this cohort in understanding these less explored *AFF2* repeat sizes and newly associated phenotypes.

## Introduction

The most prevalent form of non-syndromic X-linked intellectual disability (NS-XLID), the Fragile XE Mental Retardation Syndrome (MIM #309548), with an estimated frequency of 1/50 000 newborn males, is caused by a GCC expansion above 200 triplet repeats (full mutation) at the *AFF2* gene 5′ UTR^1–6^. Normal individuals show alleles with 6–30 GCC repeats that are stable upon transmission. Alleles with 31–60 GCC repeats are considered intermediate or grey-zone and may vary slightly upon transmission. Alleles with repeats ranging from 61 to 200 GCC repeats are considered unstable and named premutations^7,8^. When the repeats expand to over 200 GCC repeats, the so-called full mutation, gene transcription is silenced due to methylation, causing intellectual disability^9–11^. *AFF2* gene methylation is variable with reports showing that alleles with 130 GCCs can be methylated^12^. The GCC repeat can either expand or contract and is equally unstable when transmitted through both males and females. The absence of FRAXE pathognomonic features hampers early recognition, limiting testing and molecular confirmation^5,13–15^. In some patients the presence of borderline cognitive functioning hinders intellectual disability classification^16,17^. Several commercial PCR-based kits have been developed for FRAXA analysis, yet, to the best of our knowledge none exist for FRAXE testing, probably due to the scarcity of research reports as well as clinical cases^18–21^. Therefore, in some countries genetic testing for *AFF2* is considered investigational. The absence of FRAXE pathognomonic features requires careful clinical assessment, and mandates genetic testing for early recognition and appropriate monitoring^22^. Hence, our laboratory uses a low-cost multiplex PCR-based strategy to determine simultaneous allele sizing of FRAXA and FRAXE loci in both males and females that fulfil clinical criteria for Fragile X Syndrome, idiopathic intellectual disability (FXS, MIM #300624)^23^. In the presence of an expanded allele, the PCR result is usually inconclusive as the amplification of expanded alleles is inefficient in high repeat sizes. Furthermore, around 20% of female samples are uninformative due to possible homozygosity (both alleles share the same number of repeats) for one or both loci. These observations prompted us to develop a rapid triplet-primed amplification (TP-PCR) screening strategy to size the *AFF2* GCC repeat and accurately assess homozygosity in female samples. In the case of *FMR1* a commercially available TP-PCR kit is used. Overall, this study allowed validation of a homozygosity screening strategy, spotting false negatives from the multiplex-PCR allowing the identification of FRAXE-related expansions with putative diagnostic utility.

## Results

### TP-PCR development and optimization

**Figure 1:**
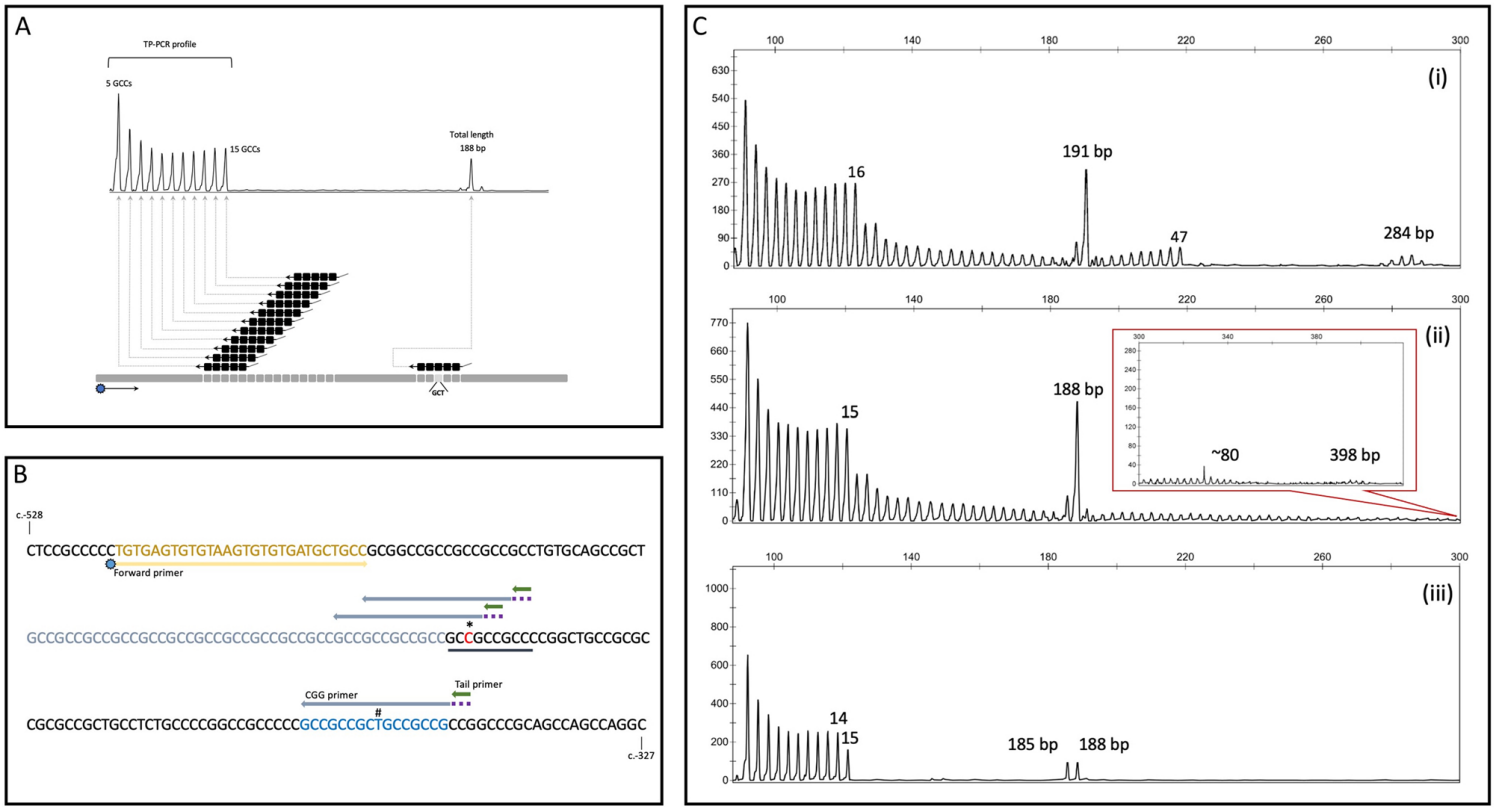
(**A**) Diagram of the TP-PCR principle. Grey squares represent tandem GCC repeats at the *AFF2* gene 5′ UTR. Black squares represent annealing of "CGG primer” within the GCC tract and in a second priming site overriding the rs1333167094 “T” SNP (when GCT is located exactly in the middle of the (CGG)5C primer sequence). Above is an electropherogram illustration after capillary electrophoresis on automatic sequencer, reflecting the total repeat length (higher rightmost peak), that corresponds to the distance between the forward primer and the sole GCC priming site 52 bp downstream of the end of the repeat. The image is not at scale; (**B**) Partial *AFF2* sequence (GRCh37/hg19, NM_002025.3) harbouring the primer binding sites. Annealing sites within the GCC repeats (light blue), generating stutters, and at (GCC)2GCT(GCC)2G sequence located 52 nucleotides downstream (dark blue). “Forward primer” and corresponding binding site (yellow); “CGG primer” (blue arrow and dashed purple line) (CGG)5C; Tail primer (green arrow) and SNPs c.-413T > C rs868949662 (*) (highest population MAF: 0.02) and c.-355T > C rs1333167094 (#) (highest population MAF: < 0.01) are also indicated. In case of rs868949662 “C”, the nucleotide following the 3rd GCC triplet is a “C” hampering its recognition, as a result instead of the three additional GCC triplets, only two are recognized (region underlined); (**C**) Examples of TP-PCR results. (i) Female heterozygous sample with normal and intermediate alleles; (ii) Female heterozygous sample with normal and expanded alleles; superimposed close-up electropherogram shows the continuous stuttering after the prominent peak with 188 bp, an indication of the presence of an expanded allele that is identified but not correctly sized by this assay; (iii) Female heterozygous sample with two normal alleles differing by one repeat (14 and 15 GCCs).

For the optimization of the TP-PCR assay conditions, we used four male and female samples, previously characterized by sequencing and Southern blot analyses (Supplementary Fig. S1). Taking advantage of the (GCC)2GCT(GCC)2C sequence located 52 nucleotides downstream of the unstable GCC tract, specific primer sequence and ligation conditions allowed the simultaneous amplification of the triplet repeat profile and the determination of the allele total length in one assay. The use of a (GCC)5 primer allows ligation to occur at (GCC)2GCT(GCC)2C, with SNP rs1333167094 “T” being unnoticed (Fig. 1). The TP-PCR electropherogram reflects the total repeat length as well as each GCC triplet, represented as a repetitive stutter pattern where the leftmost peak corresponds to repeat number 5 and each beyond that being represented as an individual peak until the end of the repeat tract is reached. The downstream peak corresponds to the distance between the forward primer and the sole GCC priming site 52 bp downstream of the end of the repeat. For instance, a peak of around 197 bp corresponds to 18 GCCs matching the thirteen (plus five triplets of the “CGG primer”) consecutive fragments differing by 3 bp (Fig. 1). Our TP-PCR assay is also able to discriminate alleles differing by a single repeat. In samples carrying larger alleles, a continuous series of uninterrupted triplet fragments is displayed, overlapping the total repeat size peak. Despite being visible triplets with an RFU greater than the height of the noise signal, only those with 30 RFUs or above are considered, allowing to accurately size expanded alleles up to 80 GCCs (Fig. 1C,ii). In cases where alleles are visible with a stuttering pattern past the established threshold of 80 repeats, further testing is mandatory following the classical/routine workflow (e.g. Southern blotting).

### TP-PCR validation

Following optimization, a total of 500 female samples, previously classified as uninformative by multiplex-PCR (due to observed homoallelism), were analyzed. In our cohort, FRAXE alleles ranged between 11 to 107 GCC triplets, in which alleles with 15 repeats are the most frequent. As expected, a homozygous normal result was confirmed by TP-PCR in the vast majority of the samples tested (n = 483; 96.6%), showing 13 different genotypes (Table 1). The most common genotype was 15/15 which is expected because the 15 GCC allele is the most common in the European population. Allele distribution is similar to that obtained by Clark et al., with the 15 (n = 692), 18 (n = 84) and 16 (n = 76) GCC alleles being the most common, followed by those with 20 GCC (n = 29) suggesting a bimodal distribution (Table 1)^8^. The unique design of the “CGG primer” in the reverse sense, with a 5′ tail and (CGG)5C at its 3′ terminus, enables the annealing anywhere within the GCC tract as well as rs1333167094 “T” SNP overriding leading to primer ligation in a second distal site. This occurs because the non-complementary “T” is located exactly in the middle of the (CGG)5 primer’s sequence. In case of rs868949662 “C”, the primer is unable to bind due to the absence of ligation in 3′ end nucleotide of the primer. We speculate that the presence of a nucleotide interspersed at the GCC stretch would likely produce a partial peak dropout as a consequence of the absence of annealing as that interspersed nucleotide is located towards the primer 3′ end. As no partial or total allele dropout was observed in our cohort, we anticipate that interspersions in the *AFF2* repetitive region are scarcer than the AGG interruption(s) in the *FMR1* gene repetitive region.

**Table 1:** Homozygous genotypes identified among the 500 female samples.

Number of GCC repeats	Number of samples	Percentage (%)
11	1	0.21
12	1	0.21
14	6	1.24
15	350	72.46
16	43	8.90
17	3	0.62
18	45	9.32
19	7	1.45
20	13	2.69
21	4	0.83
23	4	0.83
24	4	0.83
25	2	0.41

### Unexpected alleles

**Figure 2:**
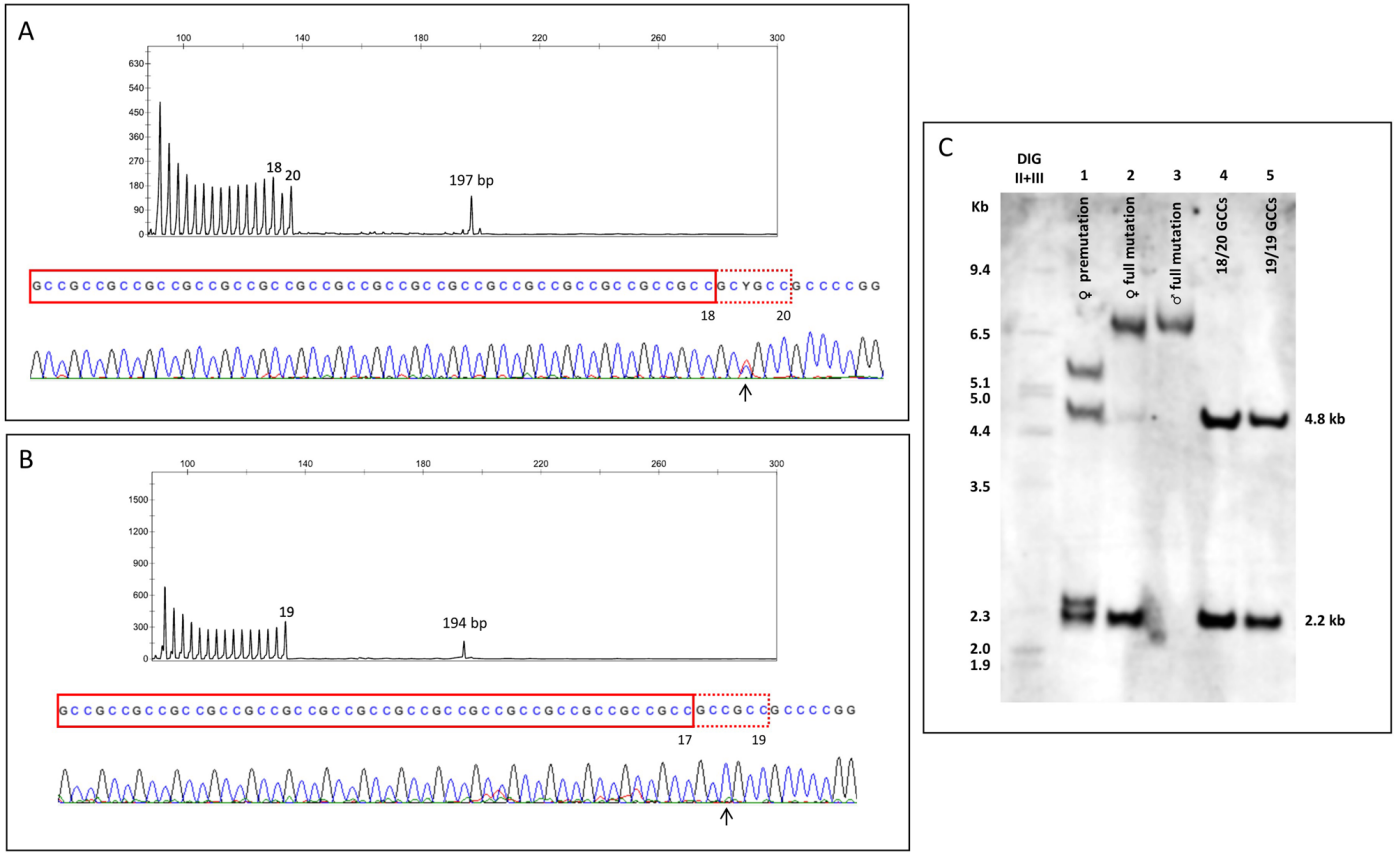
Examples of atypical results. TP-PCR electropherograms, sequencing and Southern blot of samples where TP-PCR profile sizing differs from that of the total repeat length. (**A**) Female sample showing 18 and 20 GCCs TP-PCR profile and a 197 bp total length peak; c.-413T > C heterozygous genotype (arrow); normal Southern blotting result (C-lane 4); (**B**) Female sample showing 19 GCCs and a 194 bp total length peak; c.-413T > C homozygous genotype (arrow); normal Southern result (C-lane 5); (**C**) Lane 1, premutation female sample banding pattern: normal (2.2 and 4.8 kb), premutation (2.2 kb+) and a fragment with 5.4 kb due to the loss of an AflIII restriction site (NG_016313.1g.2379G > C heterozygous, rs5980369 highest population MAF: 0.169); Lanes 2 and 3, full-mutation female and male carriers, respectively (for details see Supplementary Fig. 1); Lanes 4 and 5, Normal female samples with 18/20 GCCs and 19/19 GCCs, respectively.

The majority of normal-sized alleles were identically sized by both multiplex-PCR and TP-PCR. The discrepant result observed in one sample, multiplex-PCR (15/15) and TP-PCR (15/27) might be explained by the preferential amplification of the smallest allele when using three primer pairs (multiplexing *FMR1*, *AFF2* and *ARX* genes), as there is a 36 bp (12 GCCs) difference between the size of each allele. This bias towards the smallest allele seems to be occurring more frequently with *AFF2* than *FMR1* gene alleles (in our experience, observed even with singleplex PCR). In 6 samples a second expanded allele was observed and in 11 a second unexpected normal-sized allele could be depicted by the triplet repeat profile but not by the total repeat length fragment (Fig. 2). These results prompted sequencing of the repetitive region in those samples, which revealed that the presence of the SNP minor allele "C" at the end of the GCC region [NM_002025.3:c.-413T > C (rs868949662)], resulted in the extension of the repeat tract by three repeats. The nucleotide following the last GCC triplet is a “C”, hampering its recognition due to non-complementarity in the very last nucleotide (3′) of the primer (because of the last nucleotide, the primer needs to bind the "G" of the next triplet for amplification to occur). As a consequence, the "CGG primer” only binds to two extra repeats, without interfering in the total fragment length, and thus explaining the discrepant results. This SNP is described with a frequency of 0.021 (gnomAD), and contributes to the discriminatory power of the TP-PCR assay in heterozygous and homozygous samples carrying this variant. In samples where total length fragment shows a difference in two GCCs, when compared to TP-PCR profile, sequencing should be performed to confirm exact GCC number.

### Southern blot confirmation

Southern blotting and hybridization were performed to validate TP-PCR results, determine the exact size of expanded alleles, exclude size mosaicism and confirm methylation status. Southern blot analysis using an *AFF2*-specific probe was performed in six samples: FRAXE full mutation male sample showing a methylated fragment smear of over 6 kb (> 400 GCCs), three female samples with premutations (with 80, 84 and 107 GCCs) and two samples with intermediate alleles (with 47 and 49 GCCs) (Supplementary Fig. S2).

## Discussion

A new and accurate screening method for the analysis of the *AFF2* gene repetitive region was developed, allowing the quantification of alleles up to 100 GCCs. Among the 500 female samples, seventeen showed alleles with distinct GCC sizes of which three premutations and two intermediate-sized alleles were confirmed by Southern blotting, the remaining were confirmed to have homozygous *AFF2* alleles. The previous multiplex-PCR also failed to detect a second normal-sized allele in eleven samples (allele categorizing followed Murray et al*.* 1996)^7^. Overall, this TP-PCR assay allows a clear distinction between true homoallelism and the presence of an expanded allele and thus proves to be an accurate, reproducible, and low-cost homozygosity screening strategy. There is little information on FRAXE probably due to its rarity and/or mild phenotypic presentation that can be overlooked in the intellectually disabled population. In our cohort a 1:250 premutation carrier ratio was observed that otherwise would be overlooked as homozygous. This frequency seems quite high when compared to that of FRAXA and justifies per se the importance of such a study. *AFF2* intermediate and premutated alleles have been associated with disease due to the production of abnormal mRNA levels, similarly to other neurodegenerative disorders caused by triplet repeat expansions^24^. Association between *AFF2* and Parkinson’s disease clinical features such as gait disorder, visual hallucination and urinary incontinence have been observed, however studies with larger cohorts are needed to further understand the *AFF2* role in the disease progression and manifestation^25,26^. It also highlights the importance of the molecular characterization notably in highly heterogeneous and broad conditions such as non-syndromic intellectual disability, for proper medical care and follow-up as well as accurate genetic counselling^27^. There is a relatively high degree of variation of the FRAXE repeat size in particular, and the extensive data available from this study opens areas of research into understanding phenotype associated with relatively unexplored repeat sizes. This could be particularly interesting for the lower repeat sizes occurring with high frequency at FRAXE in this population. As the data can be linked to the newly associated phenotypes, it will provide a resource for researchers worldwide.

## Methods

Studies fully respect to the Declaration of Helsinki (1964 and amendments), the Oviedo Convention and its protocols (1997) and the Principles of good clinical practice in clinical research. Participants, legal guardians and parents/patient’s legal representatives signed informed consent for the use of DNA samples in intellectual disability research and biobanking. Genomic DNA samples had been extracted by Salting out method from peripheral blood collected in EDTA, dissolved in Tris–EDTA buffer (pH 7) and anonymously stored at 4 °C^28^. Samples were previously tested by routine FRAXA, FRAXE and *ARX* multiplex-PCR^23^. This study has been approved by DEFI—Departamento de Ensino, Formação e Investigação do Centro Hospitalar Universitário do Porto, (CHUPorto), as well as the Hospital’s Ethical Committee—N/REF.a 2018.179 (154-DEFI/153-CES) as part of Cecília Silva (CS) Master’s degree.

### Triplet-repeat primed PCR (TP-PCR) assay

The *AFF2* TP-PCR mix was prepared for a final reaction volume of 25 μL containing 1 × PCR Master Mix (Promega, Madison, WI, USA), 0.6 M Betaine (Sigma-Aldrich, St. Louis, MO, USA), 10% DMSO (Sigma-Aldrich), 0.5 × Q-Solution (Qiagen GmbH, Hilden, Germany), 0.2 mM 7-Deaza-dGTP (Roche, Basel, Switzerland), 0.2 pmol/μL forward and tail primers, 0.1 pmol/μL "GCC primer" and approximately 450 ng of gDNA. Primer sequences are as follows: (i) the “forward primer” NED labelled 5′-TGTGAGTGTGTAAGTGTGTGATGCTGCC-3′; (ii) the “CGG primer” with a nonspecific tail sequence composed by 21 nucleotides 5′-TACGCATCCCAGTTTGAGACG(CGG)5C-3′; (iii) and the “tail primer” 5′-TACGCATCCCAGTTTGAGACG-3′. Amplification cycle includes an initial denaturation step of 5 min at 98 °C, 15 cycles of denaturation at 98 °C for 60 s, annealing at 55 °C for 2 min and extension at 68 °C for 4 min, and 30 cycles at 98 °C for 60 s, 58 °C for 2 min and 68 °C for 4 min and with a final extension of 10 min at 68 °C. Five μL of TP-PCR product was added to 15 μL of a mixture (15:1) of formamide and GeneScan 500 ROX dye Size Standard (Applied Biosystems, Foster City, CA, USA), resolved on ABI PRISM 3130*xl* Genetic Analyser (Applied Biosystems) using POP-7 polymer and a 36 cm long 16-capillary array. Analyses were performed using GeneMapper Software version 4.0 (Applied Biosystems) with the NED fluorochrome peak detection threshold adjusted to 30 relative fluorescence units. A previously sequenced sample with 15 GCCs (around 188 bp in the established conditions) was used as control to determine the exact size in base pairs (bp).

### TP-PCR results validation

#### Sanger sequencing

Symmetric amplification was performed using 5′-TGTGAGTGTGTAAGTGTGTGAT-3′ and 5′-GCCCGCGCACCCAGCGAC-3′ primers, followed by asymmetric PCR using BigDye Terminator v3.1 Cycle Sequencing Kit (Applied Biosystems) according to manufacturer’s instructions and standard conditions. Products were resolved on ABI PRISM 3130*xl* Genetic Analyser (Applied Biosystems) and the results analysed using SeqScape Software version 2.5 (Applied Biosystems).

#### Southern blotting and hybridization

Around 10 μg of gDNA was digested overnight at 37 °C using endonucleases NotI (methylation sensitive) and AflIII (New England Biolabs, Ipswich, MA, USA), followed by 1% agarose gel overnight electrophoresis, blotting for 3 h and overnight hybridization at 60 °C using AFF2-AJ31Dig1 probe (GeneLink, Hawthorne, NY, USA) 1:4 diluted in Dig Easy Hyb (Roche). Dig Wash and Block Buffer Set (Roche) and CDP-Star (Roche) were used for signal revelation. Signal detection was accomplished using a Fujifilm Luminescent Image Analyzer LAS-3000 v2.2 (Fujifilm, Tokyo, Japan). DIG-labeled, DNA Molecular Weight Markers II and III (Roche), were mixed (1:1) and used as a size standard.

## Supplementary Information

Supplementary Figures.
