# Supplementary material for: Development and validation in 500 female samples of a TP-PCR assay to identify AFF2 GCC expansions
Source: Sci Rep. 2021 Jul 19;11:14676. doi: 10.1038/s41598-021-93473-5 (PMC8289994; doi:10.1038/s41598-021-93473-5)
Supplement: Supplementary file 1 — Supplementary Figures. [file 41598_2021_93473_MOESM1_ESM.docx]

Development and validation in 500 female samples of a TP-PCR assay to identify *AFF2* GCC expansions

Cecília Silva^1,2,3^, Nuno Maia^1,2,3^, Flávia Santos^1,2^, Bárbara Rodrigues^1,2^, Isabel Marques^1,2^, Rosário Santos^1,2^, and Paula Jorge^1,2^
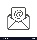


^1^Unidade de Genética Molecular, Centro de Genética Médica Jacinto de Magalhães (CGM), Centro Hospitalar Universitário do Porto (CHUPorto), Porto, Portugal;

^2^Unidade Multidisciplinar de Investigacão Biomédica (UMIB), Instituto de Ciências Biomédicas Abel Salazar (ICBAS), Laboratory for Integrative and Translational Research in Population Health (ITR), Universidade do Porto (UP), Porto, Portugal

^3^these authors contributed equally to this work


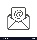
[paulajorge.cgm@chporto.min-saude.pt](mailto:paulajorge.cgm@chporto.min-saude.pt)

**Supplementary Information:**

**Figure S1.** Southern Blot restriction and banding patterns and partial GCC sequencing electropherograms. **A+B** – Double digestion with *Not*I (methylation sensitive) and *Afl*III restriction enzymes. Normal pattern, male: 2.2 kb unmethylated allele; Normal pattern, female: 4.8 kb methylated and 2.2 kb unmethylated alleles; Premutation allele, male: 2.2 kb+ fragments: 61-200 unmethylated GCC repeats; Premutation allele, female: 2.2 kb+ and 4.8 kb+ fragments: 61-200 GCC repeats; Full mutation range >200 repeats, with fully methylated CpG island: 4.8 kb++ expansion fragment. Lane 1, male sample with a full mutation; Lane 2, premutation male sample; Lane 3, premutation female sample, with an additional 500 bp (5.3 Kb) fragment due to the heterozygous NG_016313.2:g.2379G>A SNP (rs5980369) at the *Afl*III restriction site; Lane 4, normal male sample; **C** –Example of the repetitive region sequencing of a male sample with 15 GCC repeats (B - Lane 4).


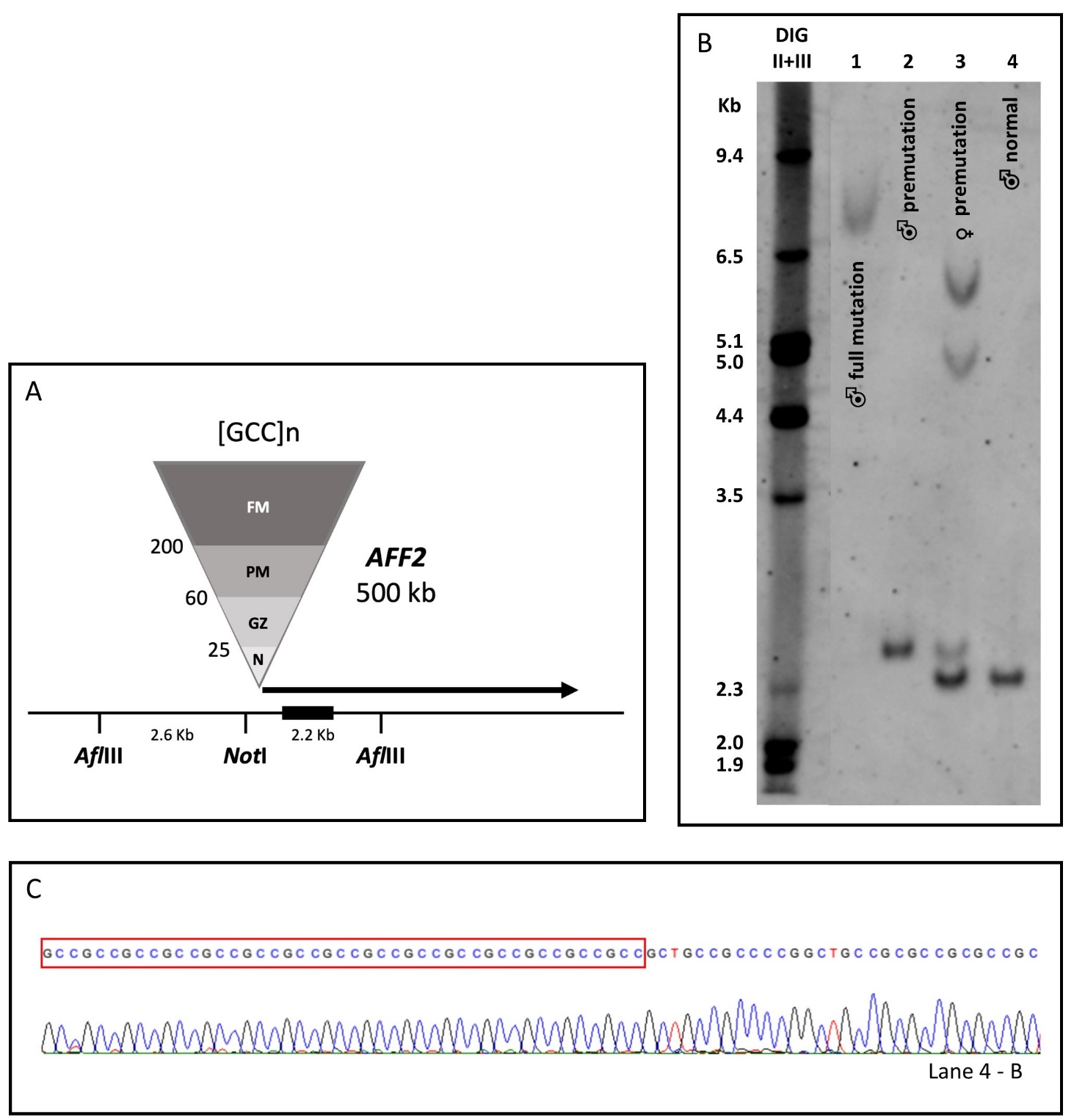


**Figure S2.** **Southern blot analysis.** Lane 1 – male sample with a full mutated allele (positive control). Lanes 2 to 6 – Female samples with respective number of GCCs indicated above each lane. The NG_016313.2:g.2379G>A SNP (rs5980369) at the *Afl*III restriction site, resulting in a normal fragment with an additional 500 bp (5.3 Kb) was detected in heterozygosity (lanes 2 to 6).

**
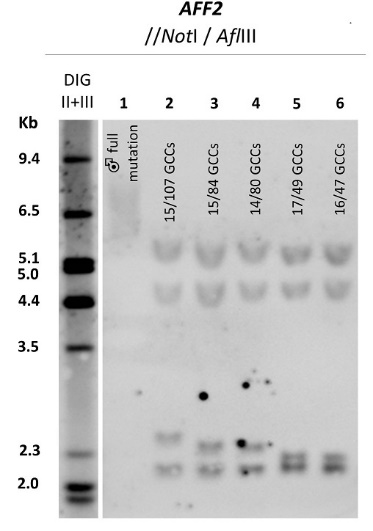
**
